# Analysis of Prognosis and Immune Microenvironment of Protein Kinase C Substrate 80K-H in Diabetic Lung Cancer Patients

**DOI:** 10.14740/wjon2663

**Published:** 2026-03-05

**Authors:** Xiang Ying Li, Yue Feng, Cun Feng Li, Hong Qiao

**Affiliations:** a Department of Endocrinology, The Fifth People’s Hospital of Shenyang City, Shenyang, Liaoning, China; b Department of Endocrinology, Second Affiliated Hospital of Harbin Medical University, Harbin, Heilongjiang, China; c Department of Rehabilitation, The Second Affiliated Hospital of Liaoning University of Traditional Chinese Medicine, Liaoning, Shenyang, China; d Department of Rehabilitation Medicine, Shaoxing Hospital of Traditional Chinese Medicine, Shaoxing, Zhejiang, China

**Keywords:** Lung cancer, Diabetes, RAGE, PRKCSH, Prognosis, Immune cell infiltration, Bioinformatics analysis

## Abstract

**Background:**

A substantial association has been established between diabetes and an elevated risk of lung cancer. This study aimed to elucidate the prognosis and characterize alterations in the immune microenvironment linked to the protein kinase C substrate 80K-H (*PRKCSH*) gene in the context of diabetic lung cancer.

**Methods:**

The expression profile of receptor for advanced glycation end products (RAGE) genes in lung adenocarcinoma (LUAD) and diabetic cohorts was analyzed utilizing data from The Cancer Genome Atlas (TCGA) and the Gene Expression Omnibus (GEO). The methodological framework included single-sample gene set enrichment analysis (ssGSEA), hallmark pathway enrichment analysis, Pearson’s correlation, and Wilcoxon tests, employing data from the Cancer Single Cell State Atlas (CancerSEA), tracking tumor immunophenotype (TIP) meta-server, and the Genomics of Drug Sensitivity in Cancer (GDSC) platform. *PRKCSH*-targeting small interfering RNA (siRNA) was synthesized and transfected into A549 cells. Functional validation of PRKCSH was conducted using real-time quantitative polymerase chain reaction (RT-qPCR), Western blotting, methylthiazolyldiphenyl-tetrazolium bromide (MTT) assays and flow cytometry.

**Results:**

The analysis identified five RAGE genes with dysregulated expression in both diabetic and LUAD conditions, which were significantly associated with the activation of signaling pathways and patterns of immune cell enrichment in diabetes. PRKCSH has been identified as an independent prognostic marker in LUAD, with associations with key biological processes such as cell cycle regulation, genomic instability responses, inflammatory mediation, and stem cell characteristics. Comprehensive pathway analysis revealed inverse relationships between PRKCSH expression and immune-related molecular mechanisms. Detailed immune profiling indicated reduced infiltration levels of various immune cell populations in association with elevated PRKCSH expression. Notably, increased PRKCSH activity in LUAD was linked to enhanced enzymatic pathway responses and greater therapeutic sensitivity to specific enzyme inhibitors. Experimental validation via gene silencing demonstrated that suppression of PRKCSH effectively reduced malignant cell proliferation while promoting apoptotic mechanisms in lung cancer models.

**Conclusions:**

This extensive investigation positioned PRKCSH as a critical prognostic biomarker and a promising therapeutic target for personalized immunotherapeutic strategies in the management of LUAD.

## Introduction

Lung malignancies constitute the primary contributor to global cancer mortality rates [1]. In recent decades, the incidence of lung adenocarcinoma (LUAD) has surpassed that of squamous cell carcinoma, establishing it as the most prevalent form of lung cancer worldwide. Currently, LUAD accounts for 50% of all diagnosed lung malignancies, and exhibits a continuous upward trend. Surgical resection remains the standard treatment for patients with stage I and II non-small cell lung cancer (NSCLC) [2]. Furthermore, novel therapeutic approaches, including chemotherapy, immunotherapy, and targeted therapies against driver mutations, are employed for patients who are not suitable for surgery [3–8]. Clinicians are increasingly encountering patients presenting with pulmonary nodules or confirmed lung carcinoma who are also diagnosed with concurrent diabetes mellitus and hyperglycemic conditions [9]. As the most widespread metabolic disorder globally, diabetes affects over 400 million individuals and has been declared a global health crisis [10]. Recent reports from the American Diabetes Association (ADA) reports rank this endocrine disorder as the fourth leading cause of mortality in the United States [11]. The Chinese population exhibits a notably high susceptibility, with a prevalence rate of 9.1%, contributing to significant public health challenges [12]. Recent evidence highlights a complex interaction between the pathophysiology of diabetes and oncogenesis across various cancer types. Specific studies have demonstrated measurable associations between diabetic status and an increased susceptibility to lung cancer development [13]. Contemporary epidemiological analyses and clinical observations consistently identify the comorbidity of diabetes and lung malignancies within patient cohorts, revealing both elevated cancer incidence rates and increased mortality metrics [14]. Comparative survival analyses indicate significantly reduced overall survival (OS) durations and cancer-specific survival rates among diabetic lung cancer patients compared to their non-diabetic counterparts. This finding suggests that diabetes may serve as an independent predictor of outcomes in lung cancer cases [15].

The receptor for advanced glycation end products (RAGE) functions as a transmembrane protein that engages with advanced glycation end products (AGEs), which are molecular byproducts formed through non-enzymatic reactions between reducing sugars and biological macromolecules such as proteins, lipids, and nucleic acids [16]. The accumulation of AGEs within cellular and tissue matrices is recognized as a significant biomarker for aging and various chronic pathological conditions. Chronic diseases such as diabetes mellitus and cancer exemplify these pathological states. Emerging research suggests that the interaction between AGEs and RAGE induces oxidative stress, thereby triggering pathways associated with cellular proliferation, angiogenesis, and inflammatory responses. This molecular interaction is considered pivotal in both the initiation and progression of several malignancies, notably lung cancer, as reported in reference [17]. Postnatally, the expression of RAGE is markedly reduced across most organ systems, with the notable exception of lung tissue. Under typical physiological conditions in adults, the pulmonary epithelium exhibits consistently high expression levels of the receptor, in stark contrast to other bodily tissues, where RAGE expression is minimal or undetectable [18–21]. The expression profile of this receptor undergoes significant changes during inflammatory pathologies, with elevated levels observed across various cell types in conditions such as diabetic complications, cardiovascular disorders, neoplastic processes, and neurodegenerative diseases [22]. Interestingly, pulmonary malignancies display an opposite regulatory pattern, with clinical studies reporting significant downregulation of RAGE in non-small cell lung carcinomas and glandular pulmonary neoplasms [23–25]. Experimental models indicate that artificially induced overexpression of RAGE in malignant pulmonary cells inhibits cell proliferation and restricts tumor growth [23, 26]. These mechanistic insights suggest that the elevated levels of AGEs in diabetic patients and the subsequent activation of the RAGE pathway may partially explain their increased susceptibility to lung malignancies, as discussed in study [27].

Protein kinase C substrate 80K-H (PRKCSH), which functions as a RAGE on cell surfaces, plays pivotal roles in various biological contexts. This protein is a critical component of the endoplasmic reticulum’s quality control system for N-glycosylation, where it is involved in the identification and degradation of misfolded proteins [28]. As the β-subunit (GluIIβ) of glucosidase II, PRKCSH’s biological importance is derived from its interactions with diverse molecular partners and its involvement in essential cellular progresses, particularly those related to protein homeostasis. Empirical evidence underscores its regulatory role in cellular activation, directed cell migration, and secretory pathways [29]. In pathological contexts, aberrant expression patterns of PRKCSH are associated with disease pathogenesis, notably in cancer. Studies have shown increased PRKCSH expression in lung cancer, with functional analyses indicating its significant impact on patient survival and tumor progression [30, 31].

This study employs computational biology techniques to examine the clinical significance of PRKCSH in patients with diabetic lung cancer, integrating multi-source genomic data from reputable repositories such as the Gene Expression Omnibus (GEO) and The Cancer Genome Atlas (TCGA). By conducting comprehensive gene expression profiling and evaluating survival patterns, the methodology demonstrates an enhanced ability to identify molecular signatures with diagnostic and prognostic relevance [32, 33]. The primary objective of the study is to identify genetic variations specific to diabetic lung cancer cohorts and assess their implications for tumor development and clinical outcomes. These findings not only establish the potential of PRKCSH as a predictive biomarker but also lay the groundwork for exploring targeted therapeutic strategies in this patient population.

## Materials and Methods

### Data acquisition and preprocessing

To investigate differentially expressed genes (DEGs) associated with diabetes, genomic data were retrieved from the GEO repository [34]. The analysis utilized the GSE15932 dataset, generated using Affymetrix Human Genome U133 Plus 2.0 Array technology, which included 16 peripheral blood samples (comprising eight diabetic patients and eight non-diabetic controls). After standardizing and applying a log2 transformation to the data, the R package “limma” was utilized to identify DEGs between groups, adhering to established thresholds of log fold-change (FC) > 0.5 and P < 0.05. Information on the advanced glycosylation end product receptor gene was retrieved from the Gene Set Enrichment Analysis (GSEA) database. For the LUAD analysis, complementary RNA-seq data comprised 516 carcinoma tissue samples and 59 adjacent tissue specimens from the TCGA-LUAD dataset, supplemented by 578 normal lung tissue profiles from the GTEx database. These datasets were integrated for a comprehensive analysis. To address inter-batch variability, batch correction for the TCGA-LUAD dataset was performed using the “combat” function from the R package “sva”.

### Correlation analysis with the pathways and immune cells

Pathway activation and immune cell infiltration patterns in diabetic specimens were quantified using single-sample gene set enrichment analysis (ssGSEA) implemented in the GSVA package [35]. Intergroup pathway score comparisons were conducted using the Wilcoxon rank-sum test, with results visualized using the “pheatmap” R package. The Hallmark gene collection (Supplementary Material 1, wjon.elmerpub.com) and the immune cell signature database (Supplementary Material 2, wjon.elmerpub.com) were utilized as reference sets for pathway and immune profiling, respectively. To elucidate the biological relevance of DEGs in the pathophysiology of diabetes, Pearson’s correlation analysis was employed to assess the associations between the expression levels of five prioritized DEGs and molecular pathway activities across eight diabetic cases.

### Prognostic value analysis of PRKCSH

The prognostic significance of PRKCSH in LUAD cases was evaluated through survival analysis using the Kaplan–Meier method, accompanied by log-rank testing. The study focused on two critical endpoints: OS and disease-specific survival (DSS). Statistical analyses were conducted using the “survival” and “survminer” R packages, while graphical representations were generated using “ggplot2”. Prognostic assessments quantified risk using hazard ratios (HRs) and 95% confidence intervals (CIs) for the DEGs. Additionally, multivariate Cox regression modeling was performed on both the TCGA-LUAD and GEO-derived GSE26939 datasets to complement the analysis. In this study, comprehensive survival modeling was conducted, incorporating clinical parameters such as patient age, sex, tumor staging (T/N/M classification), histological differentiation, and PRKCSH expression levels, to identify independent prognostic indicators for OS and DSS outcomes.

### Analysis related to tumor phenotype

The Cancer Single Cell State Atlas (CancerSEA) [36] was utilized to investigate the association between PRKCSH expression and the biological mechanisms of LUAD. This resource offers an extensive map of single-cell functional states across various malignancies, documenting 14 cellular processes, including stem-like properties, invasive capacity, metastatic potential, proliferative activity, epithelial-mesenchymal transition, vascular formation, programmed cell death, cell cycle, cellular differentiation, genomic instability, DNA repair, hypoxia, inflammatory response, and quiescence, derived from 41,900 individual tumor cells across 25 cancer subtypes. For the TCGA-LUAD, GSE26939, and GSE6345 datasets, the GSVA’s z-score metric in R [37] was employed to quantify the activation levels of functional pathway. Subsequent normalization of scores via scaling facilitated a systematic evaluation of gene-pathway correlations employing Pearson’s statistical method.

### Analysis of anticancer immune response

To further investigate the potential roles of PRKCSH across various stages of antitumor immunity, the tracking tumor immunophenotype (TIP) analytical platform was utilized. The cancer-immune cycle comprises seven distinct biological processes: neoantigen release, antigen processing and presentation, immune cell stimulation, lymphocyte migration, tumor infiltration, T cell-mediated recognition, and malignant cell elimination [38]. The TIP meta-server integrates the ssGSEA and CIBERSORT algorithms to systematically evaluate and visualize immune activation patterns throughout these stages using transcriptomic data. Complementing this approach, the EaSIeR computational framework implemented through the “easier” package in R, was employed to predict the efficacy of immune checkpoint inhibitors, taking a holistic view of the tumor microenvironment (TME) [39]. Higher EaSIeR scores were associated with improved efficacy of immune checkpoint blockade (ICB) treatment. Pathway enrichment analysis, incorporating Kyoto Encyclopedia of Genes and Genomes (KEGG) and Hallmark gene sets, were conducted for LUAD samples.

### Drug sensitivity analysis

To investigate the association between PRKCSH expression and chemotherapeutic response, we conducted an extensive study utilizing datasets from the Genomics of Drug Sensitivity in Cancer (GDSC) and the Connectivity Map (cMAP). Our analytical approach employed the “pRRophetic” R package, which integrated statistical significance and effect size to assess drug sensitivity.

### Cell culture and treatment

A549 human lung carcinoma cells (C6053) were sourced from Beyotime (Shanghai, China). These cells were cultured in Dulbecco’s modified Eagle’s medium (DMEM) (C0891, Beyotime) supplemented with 10% fetal bovine serum (C0232, Beyotime) and 1% antibiotic mixture (ST488S, Beyotime) under standard conditions (37 °C, 5% carbon dioxide (CO_2_)). Gene silencing was performed using Lipofectamine 3000 (L3000001, Invitrogen, USA) to transfect *PRKCSH*-targeting small interfering RNA (siRNA, GenePharma, Shanghai) and non-targeting controls. Following a 48-h post-transfection incubation period, subsequent experimental analyses were conducted.

### Real-time quantitative polymerase chain reaction (RT-qPCR)

The messenger RNA (mRNA) expression levels of *PRKCSH* in cellular samples were assessed utilizing RT-qPCR methodologies. Total RNA was isolated from A549 cells employing the TRIzol reagent (15596018, Invitrogen), and subsequently reverse transcribed into cDNA using the Bio-Rad ScripTM cDNA Synthesis Kit (1708890, Bio-Rad, Hercules, CA, USA), in accordance with the manufacturer’s instructions. Amplification reactions were performed in 20-µL volumes, comprising 1 µL of complementary DNA (cDNA) template, 1 µL each of 10 µM forward and reverse primers, 10 µL of 2 × SYBR Green PCR Mastermix (SR1110, Solarbio, Beijing, China), and 8 µL of DEPC-treated water, and were processed using Bio-Rad CFX Manager software. Primer sequences were as follows: PRKCSH-F 5′-GGCGTCTCCCTCACCAATCATC-3′, PRKCSH-R 5′-TCTCCTCCCGTGCCTTCTTCCAGT-3′, GAPDH-F 5′-ATCATCCCTGCCTCTACTGG-3′, and GAPDH-R 5′-TGGGTGTCGCTGTTGAAGTC-3′.

### Western blotting

Total proteins were extracted from tumor cells using radio immunoprecipitation assay (RIPA) lysis buffer (20101ES60, YEASEN, Shanghai, China). Protein quantification was conducted with the bicinchoninic acid (BCA) Protein Assay Kit (20201ES76, YEASEN), followed by the separation of 20 µg samples via 10% sodium dodecyl sulfate polyacrylamide gel electrophoresis (SDS-PAGE) electrophoresis. The separated proteins were then transferred to polyvinylidene fluoride (PVDF) membranes (36126ES03, YEASEN) using standard electroblotting techniques. The membranes were subjected to blocking with a bovine serum albumin (BSA) solution for 60 min at ambient temperature, followed by overnight incubation at 4 °C with primary antibodies: PRKCSH (dilution 1:10,000, PA5-21398, Invitrogen) and glyceraldehyde-3-phosphate dehydrogenase (GAPDH) (dilution 1:5,000, MA5-15738, Invitrogen). After three washes with tris-buffered saline containing Tween (TBST) washes, the membranes were incubated with horseradish peroxidase (HRP)-conjugated goat anti-rabbit IgG secondary antibodies (dilution 1:10,000, 31402, Invitrogen) for 60 min at room temperature. Protein visualization was conducted using the BeyoECL Plus kit (P0018S, Beyotime), and the quantitative analysis of band intensities was performed using Image-ProPlus software (Media Cybernetics, Inc., Rockville, MD, USA).

### Methylthiazolyldiphenyl-tetrazolium bromide (MTT) analysis

A549 cells were harvested 48 h post-transfection, and seeded into 96-well plates at a density of 5 × 10^4^ cells per well, maintained under 5% CO_2_ at 37 °C. After a 24-h incubation, 10 µL of MTT reagent (C0009S, Beyotime) was added to each well for a 4-h incubation. Following the removal of the supernatant, 100 µL of dimethyl sulfoxide (ST038, Beyotime) was added to dissolve the formazan crystals. Optical density measurements were performed at a wavelength of 570 nm using a microplate reader (Thermo Fisher Scientific, Waltham, MA, USA).

### Flow cytometry

Apoptosis in A549 cells was assessed via flow cytometry using the Annexin V-fluorescein isothiocyanate (FITC) Apoptosis Detection Kit (CA1020, Solarbio). Following washing with ice-cold phosphate-buffered saline (PBS, P1020, Solarbio), the cells were resuspended in 1 mL of binding buffer and subsequently stained with FITC-annexin V and propidium iodide (PI) for 5 min under light-protected conditions. Quantification of cellular apoptosis was conducted using a FACScan flow cytometer (BD Biosciences, NJ, USA), with data analysis performed using BD CellQuest Pro software (version 5.1, BD Biosciences).

### Statistical analysis

All statistical analyses were executed using R software, employing the DESeq2 and “limma” packages for differential expression analysis of TCGA and GEO datasets, respectively. Wilcoxon rank-sum tests were utilized to evaluate differences in DEG expression patterns between malignant and adjacent normal tissues. Survival outcomes in patients with LUAD were depicted using Kaplan–Meier survival curves, and differences between groups were evaluated via log-rank tests. Independent prognostic factors were determined using both univariate and multivariate Cox proportional hazards regression models. Pearson’s correlation coefficients were employed to calculate correlations. Graphical representations were produced using R software (version 4.2.0) and associated online platforms. Statistical significance was established at P < 0.05 for all experimental analyses.

Institutional Review Board approval was not applicable to this study, and ethical compliance with human and animal studies was not applicable.

## Results

### Selection of potential target genes

**Figure 1:**
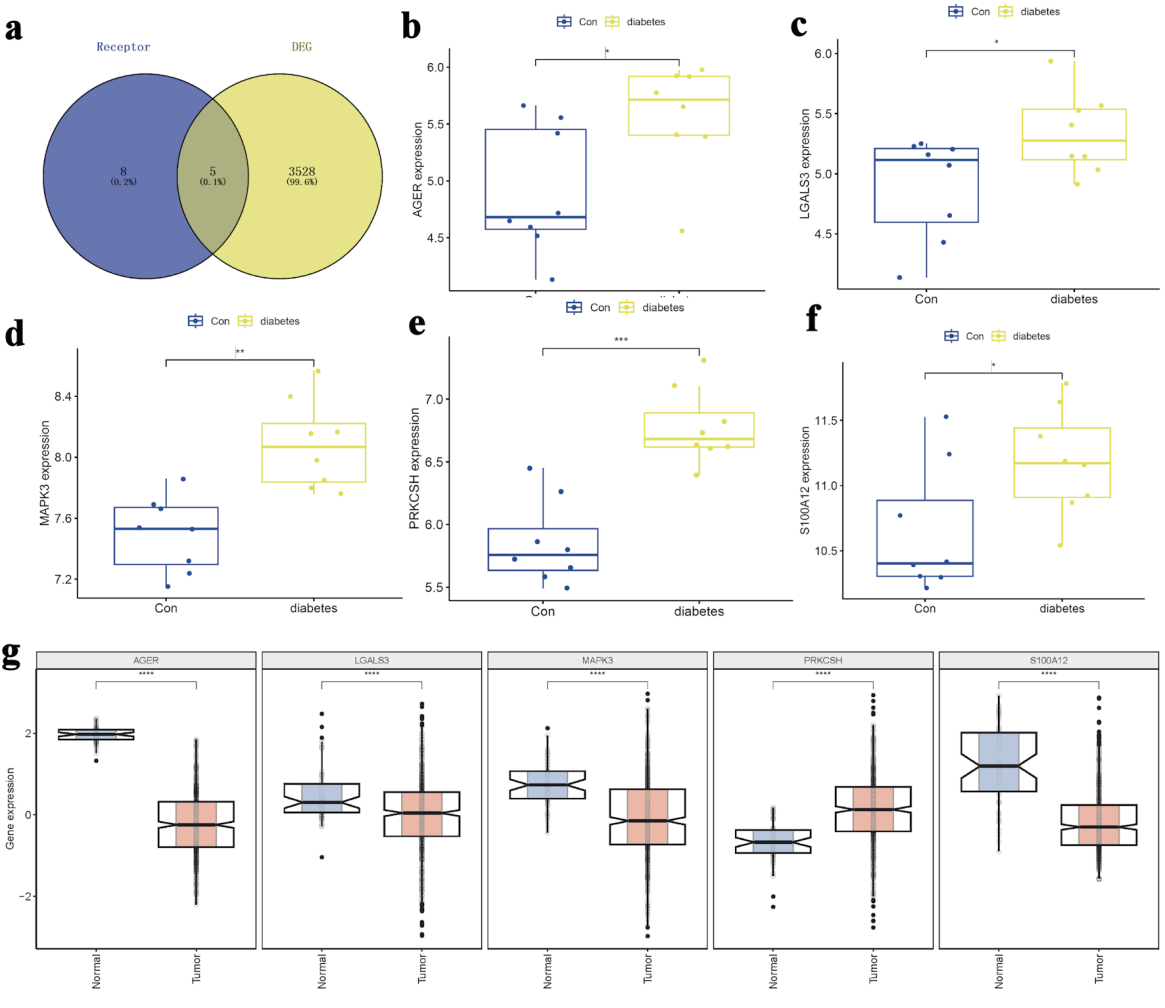
Selection of candidate target genes. (a) Intersection of DEGs from the GSE15932 diabetes dataset with genes related to the advanced glycosylation end product receptor. (b–d) Comparative expression analysis of *S100A12*, *AGER*, and *PRKCSH* between diabetic and control groups. (e, f) Expression profiles of *MAPK3* and *LGALS3* across the study cohorts. (g) Differential expression patterns of *S100A12*, *AGER*, *PRKCSH*, *MAPK3*, and *LGALS3* in LUAD tissues compared to adjacent non-tumorous and normal lung tissues. AGER: advanced glycosylation end-product-specific receptor; DEGs: differentially expressed genes; LGALS3: galectin-3; LUAD: lung adenocarcinoma; MAPK3: mitogen-activated protein kinase 3; PRKCSH: protein kinase C substrate 80K-H.

To identify potential target genes, the “limma” package in the R programming environment was utilized for differential expression analysis of the GSE15932 dataset from the GEO repository. By applying selection criteria of logFC > 0.5 and P < 0.05, a total of 3,533 candidate genes were identified within the GSE15932 dataset. Simultaneously, 13 genes related to advanced glycosylation end product receptors were extracted from the GSEA database. Venn diagram analysis identified five overlapping DEGs (*S100A12*, advanced glycosylation end-product-specific receptor (*AGER*), *PRKCSH*, mitogen-activated protein kinase 3 (*MAPK3*), and galectin-3 (*LGALS3*)) (Fig. 1a). The comparative expression analysis revealed significantly elevated levels of these DEGs in diabetic samples relative to control samples, as depicted in the box plots (Fig. 1b–f). Subsequent analysis examined the expression profiles of these genes among LUAD tissues, adjacent non-cancerous tissues, and normal pulmonary specimens. In LUAD tissues, the expression of PRKCSH was significantly higher compared to adjacent normal lung tissues, whereas other markers exhibited opposite trends (Fig. 1g).

### Analysis of pathway and immune cell enrichment in diabetes

**Figure 2:**
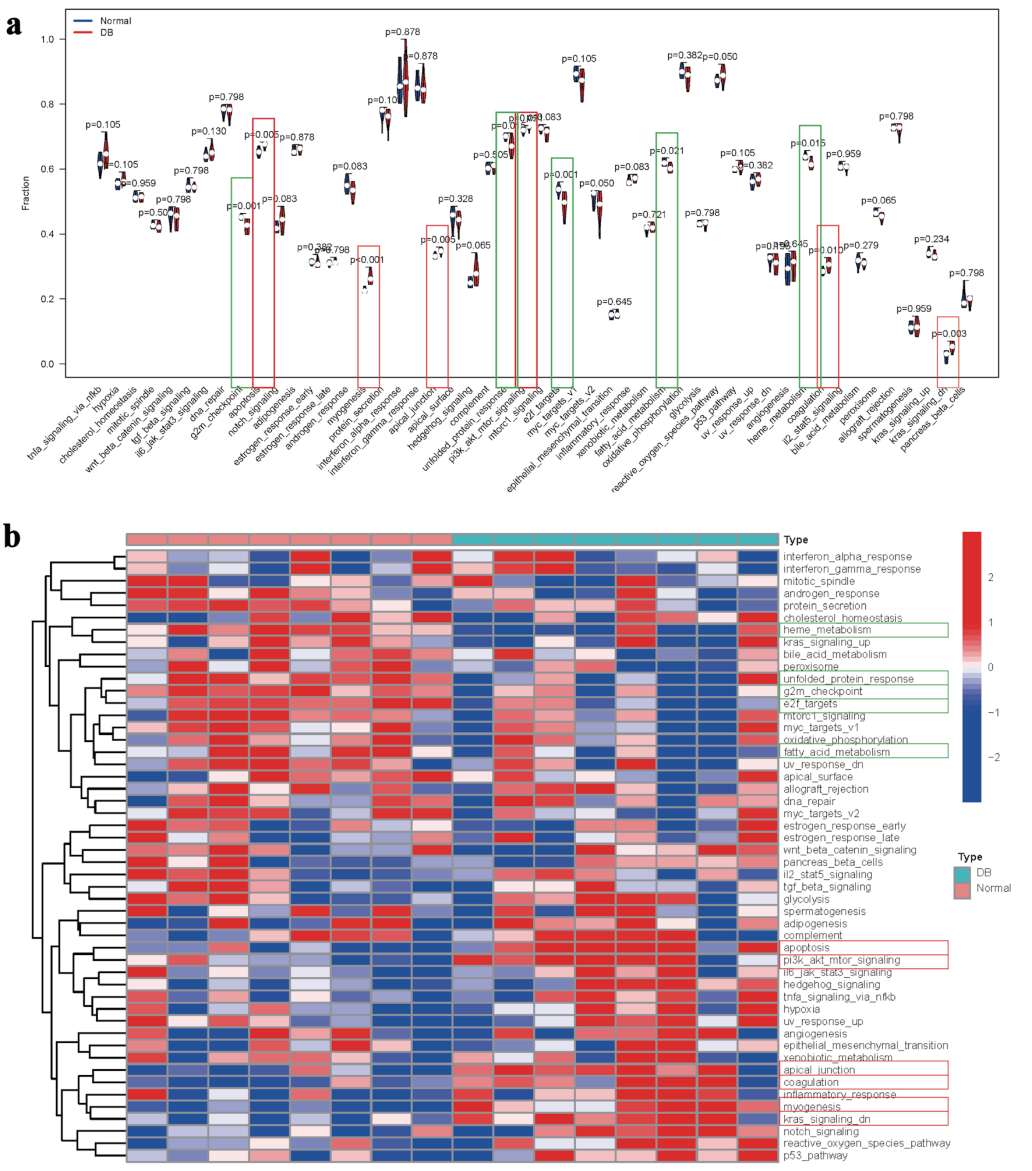
Pathway enrichment analysis in diabetes. (a) Comparative analysis of pathway enrichment scores between experimental groups. (b) Clustered heatmap visualization of pathway enrichment scores. Note: Items with the significant upregulation were marked with red rectangle, while items with the significant downregulation were marked with green rectangle.

Utilizing the reference gene set, pathway enrichment scores were calculated and compared across different groups (Fig. 2a). Diabetic specimens showed increased activity in pathways related to apoptosis, myogenesis, apical junction, phosphoinositide-3 kinase/protein kinase-B/mammalian target of rapamycin (PI3K/AKT/mTOR) signaling, coagulation, along with downregulated KRAS signaling. In contrast, reduced scores were observed for pathway associated with the G2M checkpoint, unfolded protein response, E2F targets, fatty acid metabolism, and heme metabolism (Fig. 2a). Comparative pathway activation patterns among the groups were visually represented through hierarchical clustering in the heatmap (Fig. 2b).

**Figure 3:**
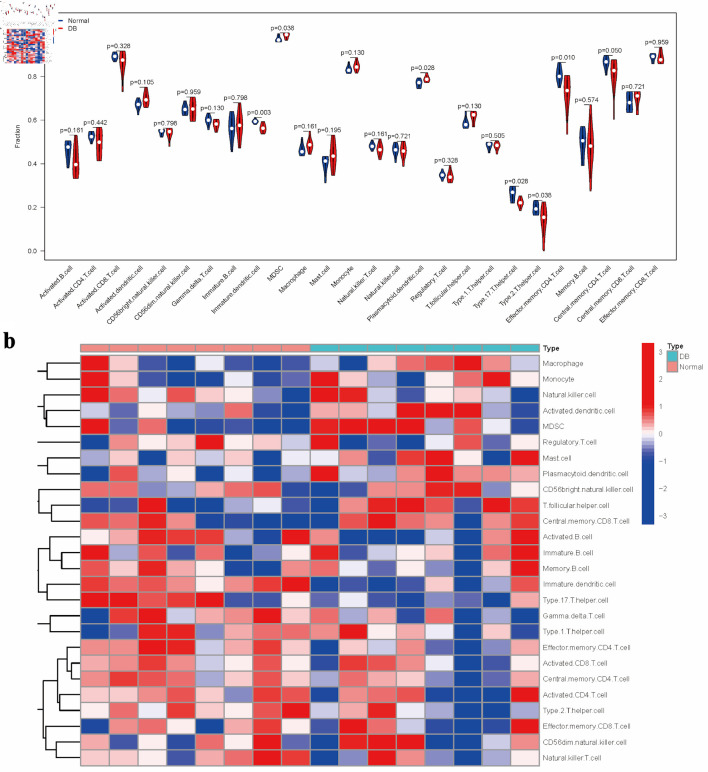
Evaluation of immune cell distribution patterns in diabetic conditions. (a) Comparative analysis of immune cell enrichment metrics across experimental groups. (b) Heatmap illustrating group-specific variations in immune cell activation levels.

Building upon the reference gene set, immune cell enrichment scores were calculated for each diabetic specimen, followed by a comparative analysis between groups (Fig. 3a). The analysis indicated that diabetic samples exhibited significantly elevated levels of myeloid-derived suppressor cells (MDSCs) and plasmacytoid dendritic cells (DC). In contrast, there were notable reductions in immature DC, type 17 T helper cell, type 2 T helper cells, and effector memory CD4 T cell (Fig. 3a). The distinct pathway enrichment patterns between the groups were illustrated using a heatmap (Fig. 3b).

**Figure 4:**
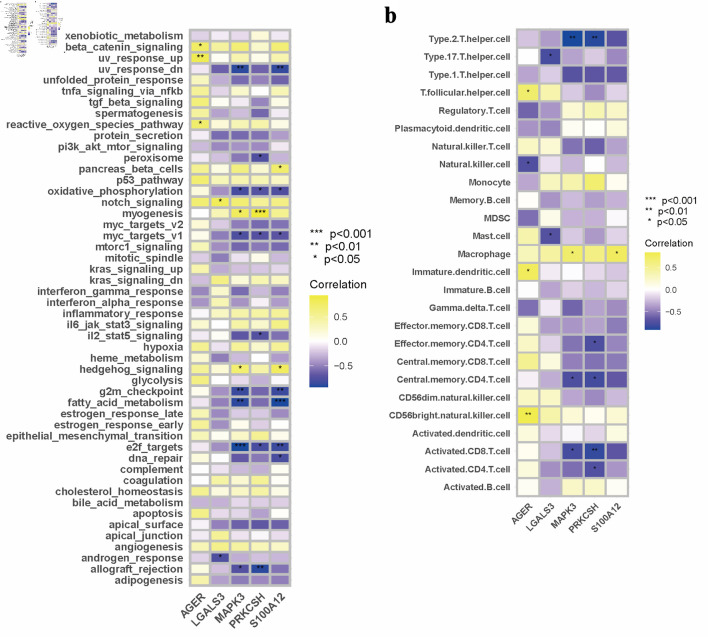
Interconnection analysis of biological pathways, immune cell populations, and gene expression characteristics. (a) A pathway–gene expression correlation network mapping. (b) An interaction matrix between immune cell composition and gene expression.

Subsequently, we explored the associations between the expression patterns of five selected DEGs and molecular pathway activities across eight diabetic specimens using Pearson’s correlation analysis. In terms of molecular pathway interactions, AGER expression was positively correlated with the activation of Wnt/β-catenin signaling activation, upregulated ultraviolet (UV) response mechanisms, and reactive oxygen species pathways in diabetic samples (Fig. 4a). The transcriptional activity of LGALS3 was positively correlated with Notch signaling transduction, while exhibiting an inverse relationship with androgen-responsive pathways (Fig. 4a). The expression of MAPK3 showed positive correlations with myogenic differentiation processes and hedgehog signaling cascades, but revealed negative associations with downregulated UV responses, mitochondrial oxidative metabolism, myc-regulated targets (v1), cell cycle G2/M checkpoints, lipid metabolism pathways, E2F-mediated transcriptional programs, and transplant rejection mechanisms (Fig. 4a). The expression patterns of PRKCSH were positively correlated with muscle development pathways, yet inversely related to peroxisomal functions, energy metabolism via oxidative phosphorylation, and interleukin 2/signal transducer and activator of transcription 5 (IL2/STAT5) signaling networks. In diabetic specimens, the expression of S100A12 demonstrated positive correlations with pancreatic β-cell function and hedgehog signaling pathways, while showing inverse relationships with suppressed UV response, mitochondrial energy metabolism, myc target activation, cell cycle progression (G2M phase), lipid oxidation processes, E2F-regulated genes, and genomic stability mechanisms (Fig. 4a). The transcriptional activity of the protein demonstrated significant associations with various biological pathways. Specifically, it was found to enhance developmental signaling cascades while attenuating pathways related to stress response pathways, proliferative markers, metabolic regulators, and DNA damage repair systems in pancreatic tissue analyses (Fig. 4a).

An analysis of immune cell proportions indicated that AGER expression was positively correlated with T follicular helper cells, immature DCs, and CD56 bright natural killer cells in diabetic samples. Conversely, a negative correlation was observed between AGER expression levels and natural killer cells (Fig. 4b). In the case of LGALS3, expression levels showed a significant inverse relationship with type 17 T helper cells and mast cells within the diabetic cohort (Fig. 4b). Furthermore, MAPK3 expression was positively associated with macrophages, while exhibiting negative correlations with type 2 T helper cells, central memory CD4 T cells, and activated CD8 T cells in diabetic specimens (Fig. 4b). The expression of PRKCSH demonstrated negative correlations with various immune cell types in diabetic samples, including type 2 T helper cells, effector memory CD4 T cells, central memory CD4 T cells, activated CD8 T cells, and activated CD4 T cells (Fig. 4b). Conversely, S100A12 expression levels showed a positive correlation with macrophage presence within the diabetic sample group (Fig. 4b).

### Prognostic value of PRKCSH in LUAD patients

**Figure 5:**
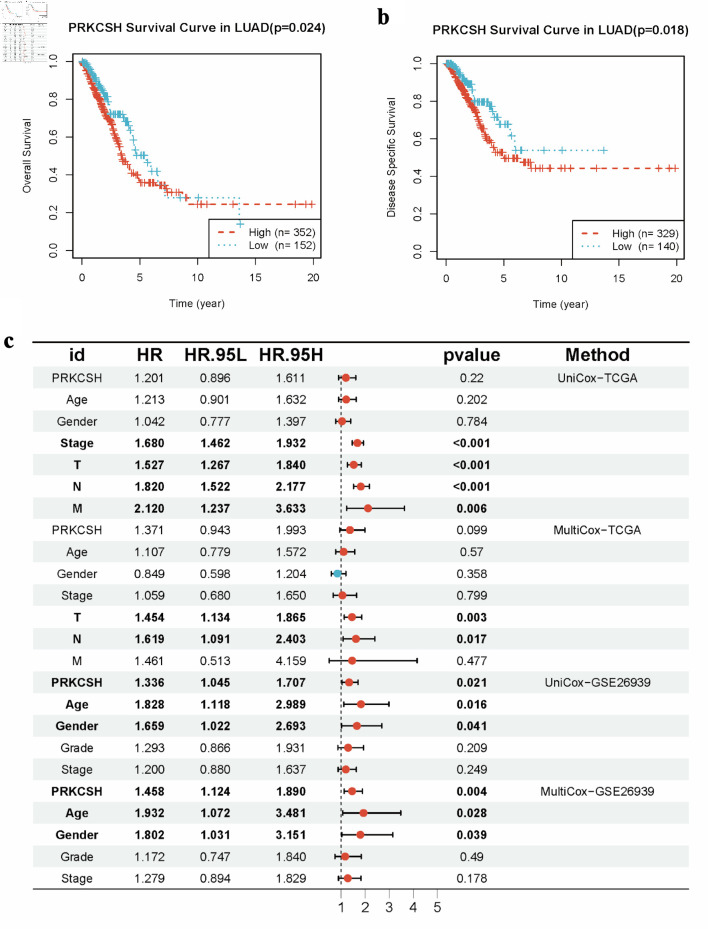
Prognostic significance of PRKCSH expression in LUAD cases. (a) Kaplan–Meier curves indicated reduced overall survival in LUAD patients with elevated PRKCSH expression. (b) Disease-specific survival analysis reveals poorer outcomes in cohorts with high PRKCSH expression. (c) Multivariate Cox regression models identify PRKCSH as an independent prognostic indicator within the TCGA-LUAD and GSE26939 cohorts. PRKCSH: protein kinase C substrate 80K-H; LUAD: lung adenocarcinoma; TCGA: The Cancer Genome Atlas; HR: hazard ratio.

Due to the similar expression patterns of PRKCSH observed across five candidate DEGs in both diabetes and LUAD contexts, this gene was selected for further single-gene analysis. To evaluate the prognostic significance of PRKCSH in LUAD, the Kaplan–Meier survival analysis was initially utilized employed using clinical data from the TCGA-LUAD cohort. Patients were stratified into PRKCSH-high and PRKCSH-low groups based on threshold values derived from the Youden index. The analytical results indicated significantly reduced OS and DSS durations in patients with high PRKCSH expression, whereas those with low expression exhibited more favorable clinical outcomes (Fig. 5a, b). Subsequent multivariate Cox regression analysis further confirmed PRKCSH as an independent predictor of OS and DSS in LUAD cases. Initial univariate analysis identified PRKCSH expression levels, patient age, and biological sex as significant correlates of survival. Subsequent multivariate analysis confirmed the independent prognostic value of PRKCSH expression. In the analysis of GSE26939 cohort, LUAD cases demonstrated elevated HRs greater than 1, indicating their significance as prognostic factors (Fig. 5c). Multivariate Cox regression modeling further validated PRKCSH as an independent prognostic indicator for LUAD, retaining statistical significance even after adjustment for conventional clinical parameters (Fig. 5c).

### Analysis related to tumor phenotype

**Figure 6:**
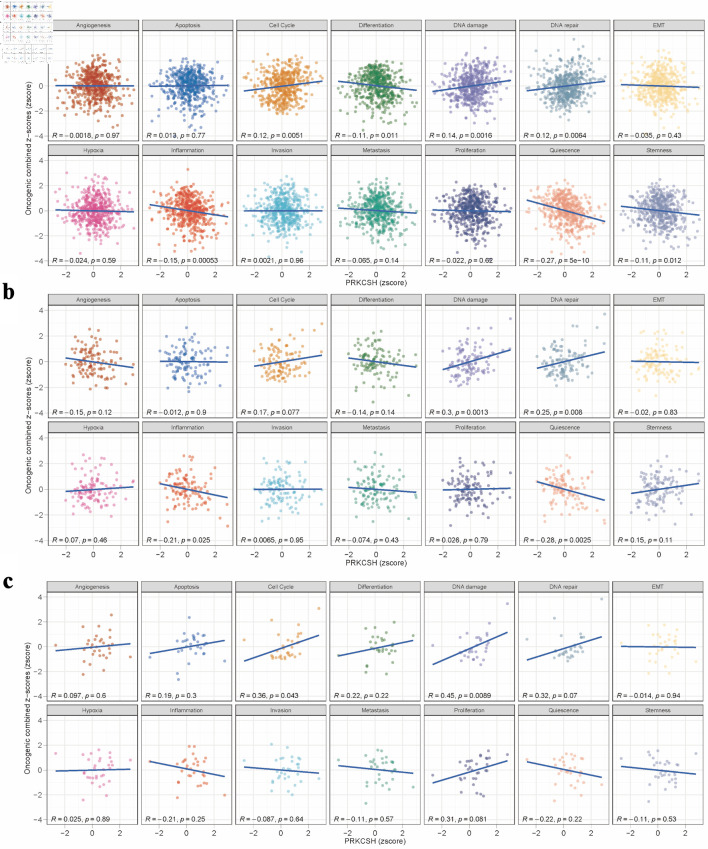
Pathway association studies of PRKCSH expression patterns. (a–c) Significant correlations between PRKCSH levels and key oncogenic processes, including cell cycle regulation, genomic stability mechanisms, cellular differentiation, inflammatory responses, and stem cell characteristics, across the TCGA-LUAD, GSE26939, and GSE63459 datasets. PRKCSH: protein kinase C substrate 80K-H; LUAD: lung adenocarcinoma; TCGA: The Cancer Genome Atlas.

Pearson correlation analysis of gene expression z-scores and GSVA scores across 14 tumor-related states revealed distinct pathway associations in different LUAD datasets. In the TCGA-LUAD cohort (Fig. 6a), PRKCSH was significantly positive correlated with proliferative pathways, including cell cycle regulation and DNA damage/repair mechanisms, while exhibiting inverse relationships with differentiation processes, inflammatory responses, cellular quiescence, and stemness characteristics. The analysis of the GSE26939 dataset (Fig. 6b) substantiates a robust association between PRKCSH and DNA maintenance pathways, specifically those involved in damage and repair, while also revealing negative correlations with inflammatory signaling and quiescent cellular states. Similarly, the examination of the GSE63459 cohort (Fig. 6c) highlights PRKCSH’s positive involvement in cell cycle progression and DNA damage response pathways.

### Analysis of anticancer immune response

**Figure 7:**
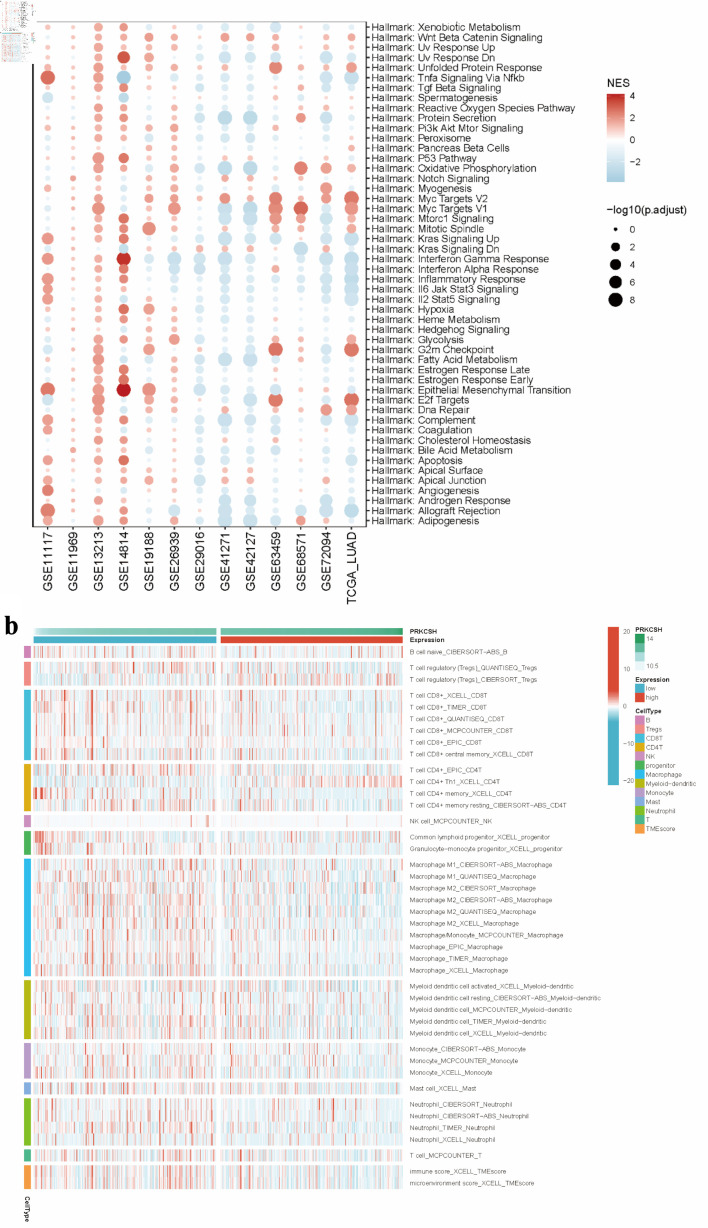
Immune microenvironment in relation to PRKCSH expression in LUAD. (a) GSEA pathway enrichment analysis using data from the TCGA-LUAD dataset. (b) Comparative immune cell infiltration patterns between subgroups with high and low PRKCSH expressions. PRKCSH: protein kinase C substrate 80K-H; LUAD: lung adenocarcinoma; GSEA: Gene Set Enrichment Analysis; TCGA: The Cancer Genome Atlas.

**Figure 8:**
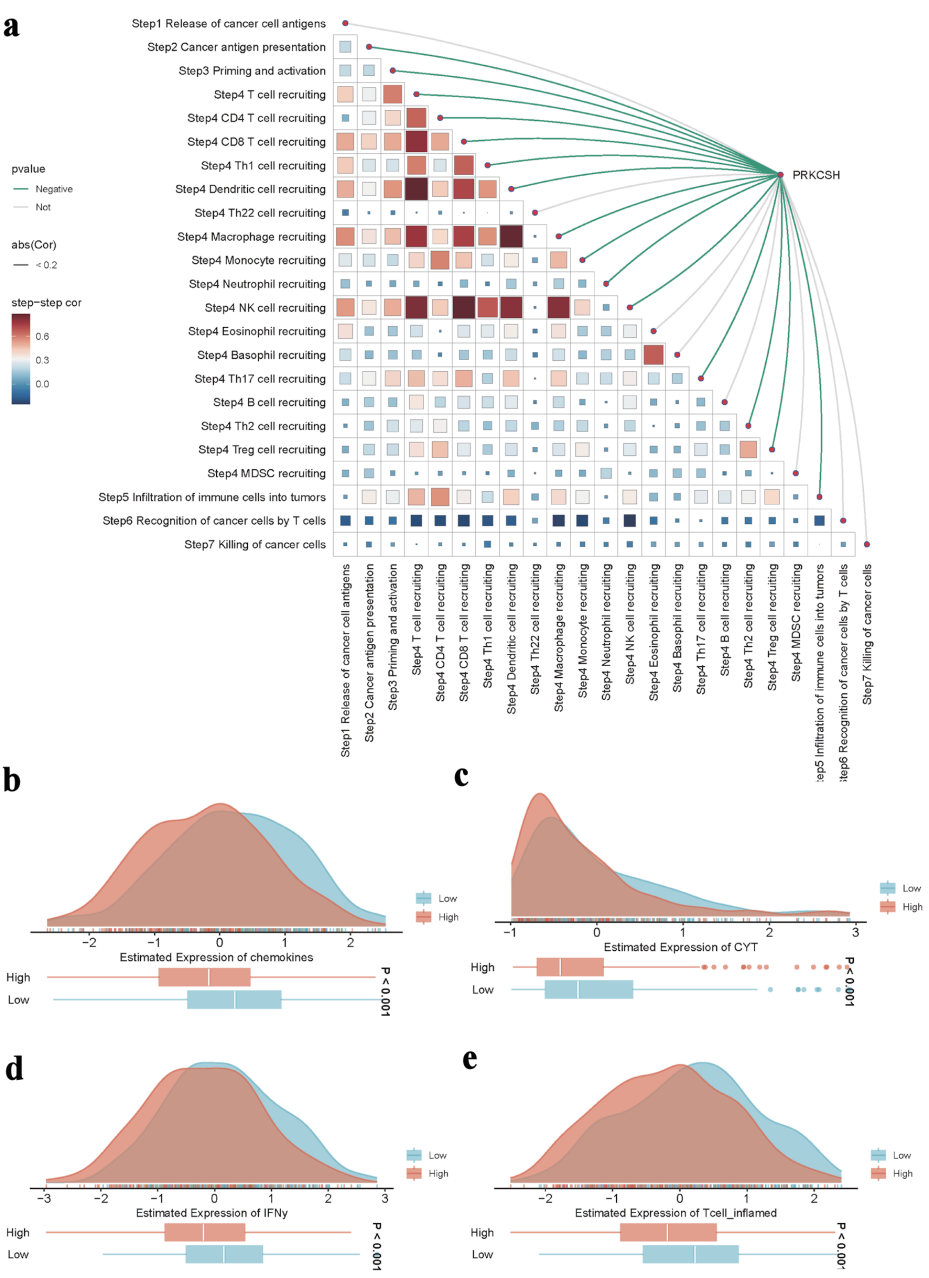
Role of PRKCSH in tumor immune regulation within LUAD. (a) The spearman correlation between the TIP score and the expression level of the *PRKCSH* gene in the TCGA-LUAD cohort. (b–e) Pathway activity comparisons related to chemokine signaling, cytotoxic potential (CYT), interferon-γ response, and inflammatory T-cell activation across different PRKCSH expression subgroups. TIP: tracking tumor immunophenotype; PRKCSH: protein kinase C substrate 80K-H; LUAD: lung adenocarcinoma; GSEA: Gene Set Enrichment Analysis; TCGA: The Cancer Genome Atlas; CYT: cytotoxic T-cell responses.

Hallmarks-GSEA analysis indicated a negative correlation between PRKCSH expression and immune-related pathways in LUAD, particularly those involving tumor necrosis factor-α-nuclear factor-κB (TNFα/NFκB) signaling, interferon (α/γ) responses, inflammatory regulation, interleukin 6-Janus kinase-signal transducer and activator of transcription 3 (IL6-JAK-STAT3) signaling, IL2-STAT5 signaling, and complement activation pathways (Fig. 7a). Stratification of LUAD patients based on median PRKCSH expression levels revealed that the low-expression subgroup maintained significant associations with immune cell infiltration patterns, thereby reinforcing its immunomodulatory role (Fig. 7b). In LUAD, Spearman’s correlation coefficients demonstrated an inverse relationship between PRKCSH and immune cell trafficking (step 4), while showing positive associations with multiple phases of the cancer immunity cycle (Fig. 8a). Subsequent stratification based on median PRKCSH expression levels facilitated a comparative analysis between subgroups. Elevated PRKCSH expression was associated with the functionality of key components of the immune microenvironment, particularly in parameters such as chemokine signaling, cytotoxic T-cell responses (CYT), interferon-γ production, and inflammatory T-cell activity (Fig 8b–e).

### Analysis of drug sensitivity

**Figure 9:**
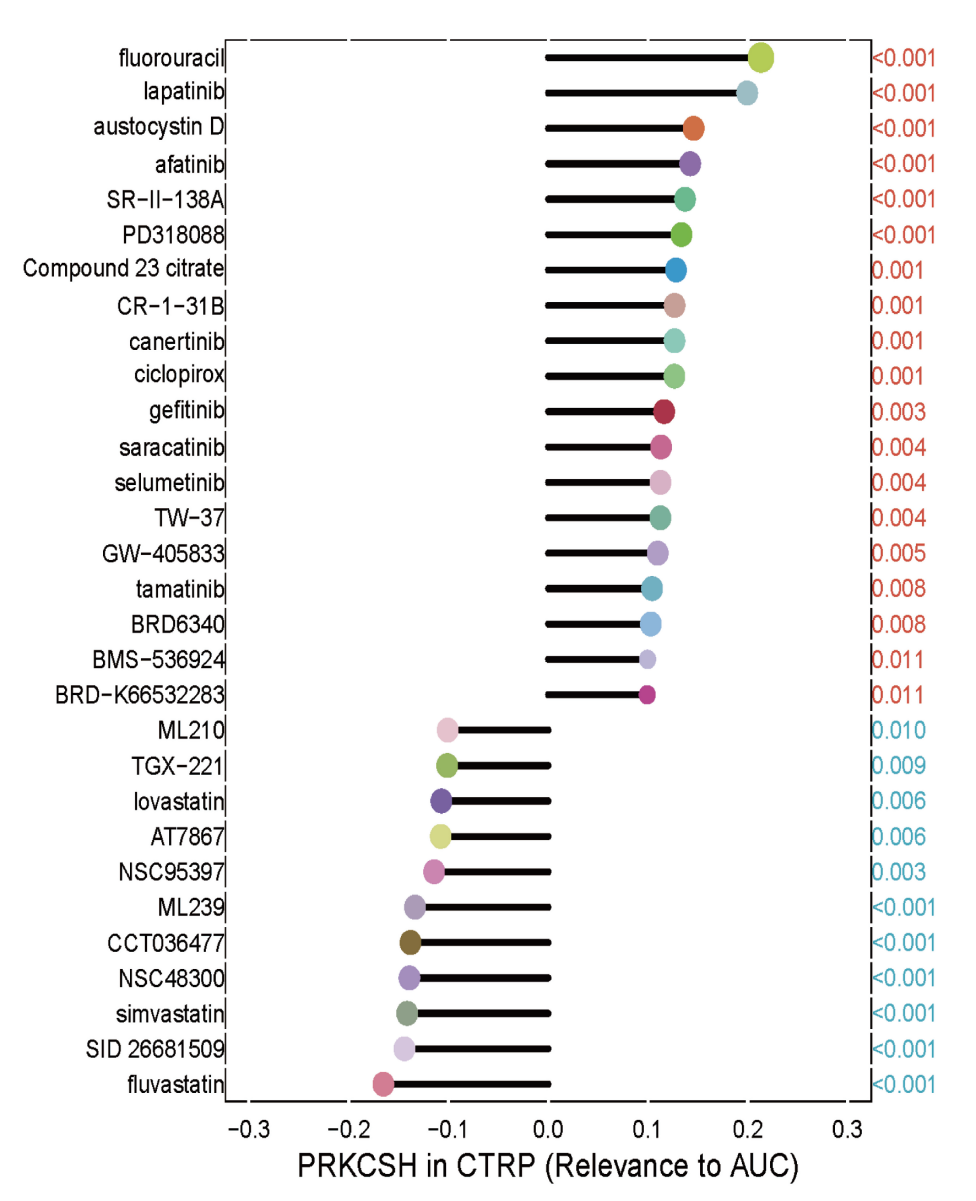
Therapeutic sensitivity correlations between PRKCSH expression and the half-maximal inhibitory concentration (IC_50_) values of pharmacological agents from the GDSC1 database. Each drug was represented by a different color, with the P value indicated in red if positively associated with the gene and in blue otherwise. The correlation coefficient was represented by the length of the bars in the lollipop plot. PRKCSH: protein kinase C substrate 80K-H; AUC: area under the curve.

Spearman’s rank correlation coefficients between gene expression profiles and pharmacological responses from the GDSC datasets revealed that increased PRKCSH expression was linked to enhanced responsiveness to enzyme inhibitors. This was particularly evident for compounds such as fluvastatin, SID 26681509, and simvastatin, which demonstrated the most pronounced inverse relationships with half-maximal inhibitory concentration (IC_50_) values (Fig. 9).

### Knockdown of PRKCSH inhibited the cell viability and promoted the apoptosis of lung cancer cells

**Figure 10:**
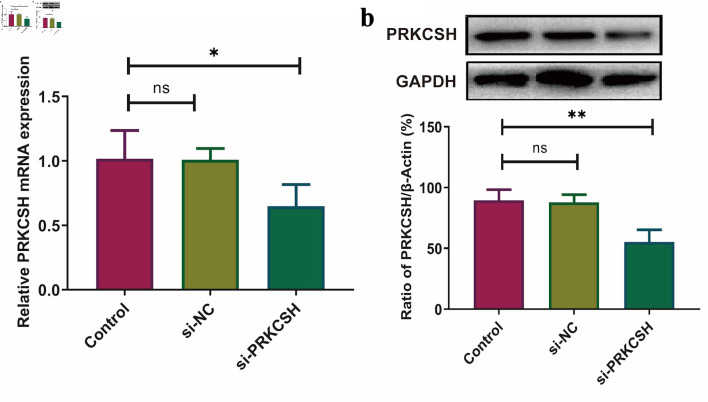
PRKCSH suppression in A549 cell models. (a) Quantitative analysis of mRNA expression using RT-qPCR, with normalization to *GAPDH*. (b) Protein level assessment via Western blotting, employing GAPDH as an internal control. RT-qPCR: real-time quantitative polymerase chain reaction; mRNA: messenger RNA; GAPDH: glyceraldehyde-3-phosphate dehydrogenase; PRKCSH: protein kinase C substrate 80K-H.

**Figure 11:**
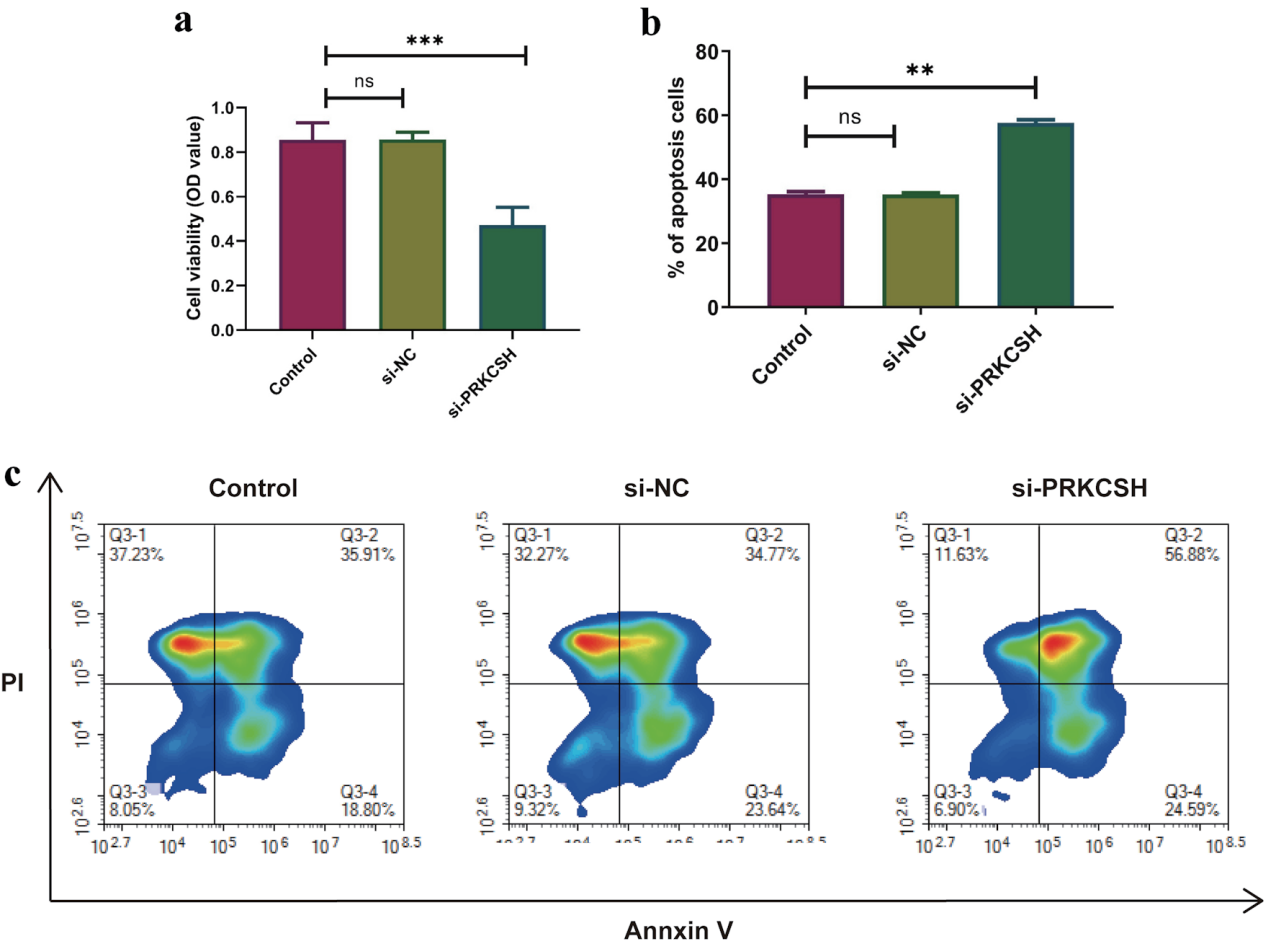
Effects of PRKCSH silencing on the dynamics of A549 cells. (a) Cell proliferation evaluation using MTT assays. (b, c) Apoptotic response quantification through flow cytometric analysis.

Considering the elevated PRKCSH expression observed in LUAD tissues, we suppressed PRKCSH levels in A549 cell lines to explore its functional significance in the pathogenesis of lung cancer. Following siRNA-mediated PRKCSH transfection, quantitative PCR and immunoblot analyses revealed significant reductions in both PRKCSH transcript abundance and protein levels (Fig. 10a, b). Genetic silencing of *PRKCSH* significantly impaired cellular proliferation capacity while simultaneously enhancing mechanisms of programmed cell death, as demonstrated by viability assays and analysis of apoptotic marker (Fig. 11a–c).

## Discussion

This study utilized computational biology approaches to identify genes associated with the RAGE that are aberrantly expressed in the context of diabetic complications and the pathogenesis of LUAD. Our analysis identified molecular intersections between these two diseases, with a particular emphasis on PRKCSH—a critical RAGE-related gene exhibiting elevated expression levels in LUAD tissues and correlating with poor patient outcomes. The oncogenic properties of PRKCSH appeared to be associated with dysregulation in essential cellular processes, including the maintenance of genomic stability, inflammatory responses, and cellular differentiation states. Comprehensive pathway analysis using Hallmarks-GSEA revealed significant suppression of immune regulatory mechanisms in tumors with high PRKCSH expression. Characterization of the TME indicated reduced infiltration of various immune cell subtypes in LUAD cases with overexpression of PRKCSH. Paradoxically, increased expression of PRKCSH was associated with enhanced activation of immune-related signaling pathways and improved therapeutic responses to specific enzymatic inhibitors. Experimental validation using gene-silencing approaches demonstrated that PRKCSH knockdown effectively inhibited malignant cellular behaviors *in vitro* models.

Pathway enrichment analysis revealed that diabetic samples exhibited a significantly higher proportion of pathways related to apoptosis, myogenesis, apical junction, PI3K/AKT/mTOR signaling, coagulation, and downregulated KRAS signaling. Conversely, these samples showed a significantly lower proportion of pathways involved in the G2M checkpoint, unfolded protein response, E2F targets, fatty acid metabolism, and heme metabolism. Apoptosis is critically implicated in the progression of type 2 diabetes, with β-cell apoptosis being a hallmark of the disease in both animal models [40] and humans [41, 42]. The PI3K/Akt/mTOR pathway plays a crucial role in regulating cell growth, cellular aging, apoptosis, glucose, protein, and lipid metabolisms [43, 44]. Dysregulation of the PI3K/AKT/mTOR pathway is associated with various diseases, including diabetes [45]. Reduced activation of PI3K/AKT/mTOR signaling has been shown to prevent the onset of diabetes mellitus [46]. Epidemiological research has demonstrated that individuals with type 2 diabetes frequently exhibit elevated levels of triglycerides and fatty acids [47–49]. Furthermore, fatty acids play a critical role in various aspects of the diabetic process, including insulin resistance, diacylglycerol biosynthesis, triglyceride accumulation, gluconeogenesis, endoplasmic reticulum stress, apoptosis, oxidative stress, and inflammation [50].

Additionally, analysis of immune cell enrichment scores revealed that diabetic samples had significantly higher proportions of MDSC and plasmacytoid DC, while exhibiting notably lower proportions of immature DC, type 17 T helper cell, type 2 T helper cells, and effector memory CD4 T cell. MDSCs have emerged as promising regulators of autoreactivity via innate immune pathways [51]. MDSC-based cellular immunotherapies represent a promising frontier in the treatment of type 1 diabetes mellitus, aiming not only to suppress immune responses but also to re-educate the immune system, thereby advancing towards durable, antigen-specific tolerance without compromising overall immune function [52]. DCs are specialized antigen-presenting cells that are pivotal in orchestrating immune responses by bridging innate and adaptive immunity. Dysregulation of DC function has been implicated in the pathogenesis of diabetes [53]. Consequently, DC-based immunomodulatory strategies warrant further investigation in diabetes research [54]. It is well established that naive CD4^+^ T cells can differentiate into various T helper cell lineages, including Th1, Th2, Th17, and regulatory T (Treg) cells [55]. Th17 cells are crucial for the recruitment of neutrophils and macrophages, mediate inflammatory responses to infectious agents, and play a role in immunity against bacterial and fungal infections, as well as in the pathogenesis of autoimmune and metabolic diseases [56, 57]. The induction of Th17 cells is significantly elevated in individuals or animal models with diabetes [57, 58]. Th17 cells are implicated in the insulin-sensitizing effect, inflammation, and other processes during diabetes [59]. Th2 cells are integral in mediating both anti-inflammatory and pro-inflammatory immune responses, which are linked to glucose homeostasis, lipid profiles, and insulin resistance in diabetes [60].

Despite significant advancements in conventional treatment modalities such as surgical intervention, radiation therapy, and cytotoxic drugs, their therapeutic efficacy remains suboptimal [61]. Recent research has identified immunotherapy as a transformative approach in oncology, with immune checkpoint inhibitors emerging as a groundbreaking strategy that shows considerable promise in significantly improving long-term survival outcomes [62, 63]. PRKCSH plays a crucial role in modulating immune responses and facilitating immune escape mechanisms within TME [64]. Investigating the potential of PRKCSH as an innovative immune checkpoint target is of substantial clinical importance. Our analysis demonstrated that elevated levels of PRKCSH expression were associated with poorer clinical outcomes in cases of pulmonary carcinoma, aligning with previous research findings [31]. These observations provide evidence for PRKCSH’s dual potential as both a diagnostic biomarker and a therapeutic target, underscoring its critical involvement in oncogenesis, disease progression, and immunomodulatory pathways.

The TME is characterized by three fundamental immunological features: pathogen elimination, immunoregulatory equilibrium, and escape mechanisms [65, 66]. This complex system functions through the coordinated actions of diverse immune cell populations encompassing both adaptive and innate immunity. Within the adaptive immune components, T lymphocytes play pivotal regulatory roles, particularly CD4^+^ helper T cells, which activate cytotoxic CD8^+^ T lymphocytes and natural killer cells via cytokine signaling, and CD8^+^ T cells, which recognize malignant cells through major histocompatibility complex class I (MHC I)-mediated mechanisms [67]. Our study identified that PRKCSH maintains consistent correlations with the infiltration levels of these immune cells, emphasizing its role in modulating immune surveillance and evasion mechanisms. Pathway analysis further revealed inverse links between PRKCSH expression and immunological signaling pathways, underscoring its multifaceted impact on immune regulation within the TME.

This study elucidates the multifaceted role of PRKCSH in modulating immunotherapeutic responses and pharmacological susceptibility within LUAD. Analyses utilizing the cancer immunity cycle framework indicate that PRKCSH exerts inhibitory effects on immune cell migration while simultaneously promoting subsequent immunological processes, such as lymphocyte activation and tumor cell eradication. These seemingly contradictory findings suggest that PRKCSH differentially regulates distinct phases of the immune response. Supporting this hypothesis, reduced expression of PRKCSH is associated with enhanced functionality of key immune effectors, including CD8^+^ T cells, tumor-associated leukocytes, and pro-inflammatory T-cell subsets, which may optimize the efficacy of ICB therapies. Pharmacogenomic evaluations further revealed increased therapeutic responsiveness to enzymatic modulators—specifically fluvastatin, SID 26681509, and simvastatin—in tumors with high PRKCSH expression. Collectively, these results identify PRKCSH as a dual regulator of antitumor immunity and chemotherapeutic effectiveness.

While our findings underscore the critical biological roles and clinical significance of PRKCSH, three primary limitations warrant consideration. First, our experimental validation was confined to examining PRKCSH’s effects on cellular viability and apoptotic pathways specifically in lung cancer, indicating a need for further research into its functions across other carcinogenic mechanisms. Second, the analysis of PRKCSH in this study was primarily based on the TCGA-LUAD dataset, and the A549 cell line was selected for *in vitro* experimental validation. However, the examination of only a single type of human lung cancer cell line may have introduced bias into the results. Therefore, future studies should incorporate multiple lung cancer cell lines for both *in vitro* and *in vivo* validation. Third, comprehensive investigations are necessary to elucidate PRKCSH’s specific contributions to resistance mechanisms against statin-based therapies, including fluvastatin, SID 26681509, and simvastatin. Fourth, although computational models were employed to predict therapeutic responses to chemotherapeutic and immunotherapeutic interventions, subsequent clinical trials are essential to validate the clinical applicability of this chemotherapy resistance-associated biomarker profile. Fifth, the potential target genes analyzed in this study, including *PRKCSH*, were initially screened using the GSE15932 dataset, which comprises data from eight diabetic patients and eight non-diabetic controls, as well as advanced glycosylation end product receptor-related genes obtained from the GSEA database. Analysis relevant to PRKCSH were conducted and validated using lung cancer databases such as TCGA-LUAD, GSE26939, and GSE63459, which may suggest a significant role for PRKCSH in the context of comorbidities. However, preliminary analyses of PRKCSH might be more appropriately conducted using diabetic databases, lung cancer databases, and advanced glycosylation end product receptor-related genes datasets. Therefore, further screening and analysis of the patient population are necessary to enhance the generalizability of the findings in this study.

## Supplementary Material

Suppl 1 The reference sets used for the pathway analysis.

Suppl 2 The reference sets used for the immune cell signature analysis.

## Data Availability

The datasets generated and/or analyzed during the current study are available from the corresponding author on reasonable request.

## Abbreviations

ADA: American Diabetes Association
AGER: advanced glycosylation end-product-specific receptor
AGEs: advanced glycation end products
BCA: bicinchoninic acid
BSA: bovine serum albumin
CancerSEA: Cancer Single Cell State Atlas
cDNA: complementary DNA
CIs: confidence intervals
cMAP: Connectivity Map
CYT: cytotoxic T-cell responses
DCs: dendritic cells
DEGs: differentially expressed genes
DMEM: Dulbecco’s modified Eagle’s medium
DSS: disease-specific survival
GAPDH: glyceraldehyde-3-phosphate dehydrogenase
GDSC: Genomics of Drug Sensitivity in Cancer
GEO: Gene Expression Omnibus
GluIIβ: β-subunit of glucosidase II
GSEA: Gene Set Enrichment Analysis
HRP: horseradish peroxidase
HRs: hazard ratios
IC_50_: half-maximal inhibitory concentration
ICB: immune checkpoint blockade
IL2-STAT5: interleukin 2/signal transducer and activator of transcription 5
IL6-JAK-STAT3: interleukin 6/Janus kinase/signal transducer and activator of transcription 3
KEGG: Kyoto Encyclopedia of Genes and Genomes
LGALS3: galectin-3
LUAD: lung adenocarcinoma
MAPK3: mitogen-activated protein kinase 3
MDSC: myeloid-derived suppressor cells
MHC I: major histocompatibility complex class I
mRNA: messenger RNA
MTT: methylthiazolyldiphenyl-tetrazolium bromide
NSCLC: non-small cell lung cancer
OS: overall survival
PI: propidium iodide
PI3K/AKT/mTOR: phosphoinositide-3 kinase/protein kinase-B/mammalian target of rapamycin
PRKCSH: protein kinase C substrate 80K-H
PVDF: polyvinylidene fluoride
RAGE: receptor for advanced glycation end products
RIPA: radioimmunoprecipitation assay buffer
RT-qPCR: real-time quantitative polymerase chain reaction
SDS-PAGE: sodium dodecyl sulfate-polyacrylamide gel electrophoresis
siRNA: small interfering RNA
ssGSEA: single-sample gene set enrichment analysis
TBST: Tris-buffered saline containing Tween
TCGA: The Cancer Genome Atlas
TIP: tracking tumor immunophenotype
TME: tumor microenvironment
TNFα/NFκB: tumor necrosis factor-α/nuclear factor-κB
UV: ultraviolet

## References

[1] Bray F, Ferlay J, Soerjomataram I, Siegel RL, Torre LA, Jemal A (2018). Global cancer statistics 2018: GLOBOCAN estimates of incidence and mortality worldwide for 36 cancers in 185 countries. CA Cancer J Clin.

[2] Liu Q, Medina HN, Rodriguez E, Jacobs KT, Brown C, Koru-Sengul T, Lopes G (2024). Trends and disparities in curative-intent treatment for early-stage non-small cell lung cancer: a population-based analysis of surgery and SBRT. Cancer Epidemiol Biomarkers Prev.

[3] Alduais Y, Zhang H, Fan F, Chen J, Chen B (2023). Non-small cell lung cancer (NSCLC): A review of risk factors, diagnosis, and treatment. Medicine (Baltimore).

[4] Rizzo A, Dall'Olio FG, Altimari A, Giunchi F, Ardizzoni A (2021). Role of PD-L1 assessment in advanced NSCLC: does it still matter?. Anticancer Drugs.

[5] Vitale E, Rizzo A, Maistrello L, Guven DC, Massafra R, Mollica V, Monteiro FSM (2024). Sex differences in adverse events among cancer patients receiving immune checkpoint inhibitors: the MOUSEION-07 systematic review and meta-analysis. Sci Rep.

[6] Guven DC, Erul E, Kaygusuz Y, Akagunduz B, Kilickap S, De Luca R, Rizzo A (2023). Immune checkpoint inhibitor-related hearing loss: a systematic review and analysis of individual patient data. Support Care Cancer.

[7] Bas O, Sahin TK, Karahan L, Rizzo A, Guven DC (2025). Prognostic significance of the cachexia index (CXI) in patients with cancer: A systematic review and meta-analysis. Clin Nutr ESPEN.

[8] Sahin TK, Ayasun R, Rizzo A, Guven DC (2024). Prognostic value of neutrophil-to-eosinophil ratio (NER) in cancer: a systematic review and meta-analysis. Cancers (Basel).

[9] Leiter A, Charokopos A, Bailey S, Gallagher EJ, Hirsch FR, LeRoith D, Wisnivesky JP (2021). Assessing the association of diabetes with lung cancer risk. Transl Lung Cancer Res.

[10] Zimmet P, Alberti KG, Magliano DJ, Bennett PH (2016). Diabetes mellitus statistics on prevalence and mortality: facts and fallacies. Nat Rev Endocrinol.

[11] Tomkins M, Lawless S, Martin-Grace J, Sherlock M, Thompson CJ (2022). Diagnosis and management of central diabetes insipidus in adults. J Clin Endocrinol Metab.

[12] Yang L, Shao J, Bian Y, Wu H, Shi L, Zeng L, Li W (2016). Prevalence of type 2 diabetes mellitus among inland residents in China (2000-2014): a meta-analysis. J Diabetes Investig.

[13] Kim DS, Scherer PE (2021). Obesity, diabetes, and increased cancer progression. Diabetes Metab J.

[14] Luo J, Hendryx M, Qi L, Ho GY, Margolis KL (2016). Pre-existing diabetes and lung cancer prognosis. Br J Cancer.

[15] Tseng CH (2014). Diabetes but not insulin increases the risk of lung cancer: a Taiwanese population-based study. PLoS One.

[16] Twarda-Clapa A, Olczak A, Bialkowska AM, Koziolkiewicz M (2022). Advanced Glycation End-Products (AGEs): formation, chemistry, classification, receptors, and diseases related to AGEs. Cells.

[17] Yamagishi S, Matsui T, Fukami K (2015). Role of receptor for advanced glycation end products (RAGE) and its ligands in cancer risk. Rejuvenation Res.

[18] Brett J, Schmidt AM, Yan SD, Zou YS, Weidman E, Pinsky D, Nowygrod R (1993). Survey of the distribution of a newly characterized receptor for advanced glycation end products in tissues. Am J Pathol.

[19] Katsuoka F, Kawakami Y, Arai T, Imuta H, Fujiwara M, Kanma H, Yamashita K (1997). Type II alveolar epithelial cells in lung express receptor for advanced glycation end products (RAGE) gene. Biochem Biophys Res Commun.

[20] Englert JM, Hanford LE, Kaminski N, Tobolewski JM, Tan RJ, Fattman CL, Ramsgaard L (2008). A role for the receptor for advanced glycation end products in idiopathic pulmonary fibrosis. Am J Pathol.

[21] Hanford LE, Fattman CL, Shaefer LM, Enghild JJ, Valnickova Z, Oury TD (2003). Regulation of receptor for advanced glycation end products during bleomycin-induced lung injury. Am J Respir Cell Mol Biol.

[22] Schmidt AM, Yan SD, Yan SF, Stern DM (2001). The multiligand receptor RAGE as a progression factor amplifying immune and inflammatory responses. J Clin Invest.

[23] Bartling B, Hofmann HS, Weigle B, Silber RE, Simm A (2005). Down-regulation of the receptor for advanced glycation end-products (RAGE) supports non-small cell lung carcinoma. Carcinogenesis.

[24] Schraml P, Bendik I, Ludwig CU (1997). Differential messenger RNA and protein expression of the receptor for advanced glycosylated end products in normal lung and non-small cell lung carcinoma. Cancer Res.

[25] Stav D, Bar I, Sandbank J (2007). Usefulness of CDK5RAP3, CCNB2, and RAGE genes for the diagnosis of lung adenocarcinoma. Int J Biol Markers.

[26] Kalea AZ, See F, Harja E, Arriero M, Schmidt AM, Hudson BI (2010). Alternatively spliced RAGEv1 inhibits tumorigenesis through suppression of JNK signaling. Cancer Res.

[27] Garza-Campos A, Prieto-Correa JR, Dominguez-Rosales JA, Hernandez-Nazara ZH (2023). Implications of receptor for advanced glycation end products for progression from obesity to diabetes and from diabetes to cancer. World J Diabetes.

[28] Ruddock LW, Molinari M (2006). N-glycan processing in ER quality control. J Cell Sci.

[29] Li YM, Mitsuhashi T, Wojciechowicz D, Shimizu N, Li J, Stitt A, He C (1996). Molecular identity and cellular distribution of advanced glycation endproduct receptors: relationship of p60 to OST-48 and p90 to 80K-H membrane proteins. Proc Natl Acad Sci U S A.

[30] Shin GC, Lee HM, Kim N, Seo SU, Kim KP, Kim KH (2024). PRKCSH contributes to TNFSF resistance by extending IGF1R half-life and activation in lung cancer. Exp Mol Med.

[31] Lei R, Zhou M, Zhang S, Luo J, Qu C, Wang Y, Guo P (2022). Potential role of PRKCSH in lung cancer: bioinformatics analysis and a case study of Nano ZnO. Nanoscale.

[32] Chen F, Wendl MC, Wyczalkowski MA, Bailey MH, Li Y, Ding L (2021). Moving pan-cancer studies from basic research toward the clinic. Nat Cancer.

[33] Korenjak M, Zavadil J (2019). Experimental identification of cancer driver alterations in the era of pan-cancer genomics. Cancer Sci.

[34] Barrett T, Wilhite SE, Ledoux P, Evangelista C, Kim IF, Tomashevsky M, Marshall KA (2013). NCBI GEO: archive for functional genomics data sets—update. Nucleic Acids Res.

[35] Hanzelmann S, Castelo R, Guinney J (2013). GSVA: gene set variation analysis for microarray and RNA-seq data. BMC Bioinformatics.

[36] Yuan H, Yan M, Zhang G, Liu W, Deng C, Liao G, Xu L (2019). CancerSEA: a cancer single-cell state atlas. Nucleic Acids Res.

[37] Lee E, Chuang HY, Kim JW, Ideker T, Lee D (2008). Inferring pathway activity toward precise disease classification. PLoS Comput Biol.

[38] Xu L, Deng C, Pang B, Zhang X, Liu W, Liao G, Yuan H (2018). TIP: A Web Server for Resolving Tumor Immunophenotype Profiling. Cancer Res.

[39] Lapuente-Santana O, van Genderen M, Hilbers PAJ, Finotello F, Eduati F (2021). Interpretable systems biomarkers predict response to immune-checkpoint inhibitors. Patterns (N Y).

[40] Pick A, Clark J, Kubstrup C, Levisetti M, Pugh W, Bonner-Weir S, Polonsky KS (1998). Role of apoptosis in failure of beta-cell mass compensation for insulin resistance and beta-cell defects in the male Zucker diabetic fatty rat. Diabetes.

[41] Butler AE, Janson J, Bonner-Weir S, Ritzel R, Rizza RA, Butler PC (2003). Beta-cell deficit and increased beta-cell apoptosis in humans with type 2 diabetes. Diabetes.

[42] Leonardi O, Mints G, Hussain MA (2003). Beta-cell apoptosis in the pathogenesis of human type 2 diabetes mellitus. Eur J Endocrinol.

[43] Xu F, Na L, Li Y, Chen L (2020). Roles of the PI3K/AKT/mTOR signalling pathways in neurodegenerative diseases and tumours. Cell Biosci.

[44] Yan J, Wang C, Jin Y, Meng Q, Liu Q, Liu Z, Liu K (2018). Catalpol ameliorates hepatic insulin resistance in type 2 diabetes through acting on AMPK/NOX4/PI3K/AKT pathway. Pharmacol Res.

[45] Jere SW, Houreld NN, Abrahamse H (2019). Role of the PI3K/AKT (mTOR and GSK3beta) signalling pathway and photobiomodulation in diabetic wound healing. Cytokine Growth Factor Rev.

[46] Bathina S, Gundala NKV, Rhenghachar P, Polavarapu S, Hari AD, Sadananda M, Das UN (2020). Resolvin D1 ameliorates nicotinamide-streptozotocin-induced type 2 diabetes mellitus by its anti-inflammatory action and modulating PI3K/Akt/mTOR pathway in the brain. Arch Med Res.

[47] Al-Mawali A, Al-Harrasi A, Jayapal SK, Morsi M, Pinto AD, Al-Shekaili W, Al-Kharusi H (2021). Prevalence and risk factors of diabetes in a large community-based study in the Sultanate of Oman: STEPS survey 2017. BMC Endocr Disord.

[48] Urrutia I, Martin-Nieto A, Martinez R, Casanovas-Marsal JO, Aguayo A, Del Olmo J, Arana E (2021). Incidence of diabetes mellitus and associated risk factors in the adult population of the Basque country, Spain. Sci Rep.

[49] Marchesini G, Brizi M, Bianchi G, Tomassetti S, Bugianesi E, Lenzi M, McCullough AJ (2001). Nonalcoholic fatty liver disease: a feature of the metabolic syndrome. Diabetes.

[50] Wang Y (2025). Triglycerides, glucose metabolism, and type 2 diabetes. Int J Mol Sci.

[51] Yin B, Ma G, Yen CY, Zhou Z, Wang GX, Divino CM, Casares S (2010). Myeloid-derived suppressor cells prevent type 1 diabetes in murine models. J Immunol.

[52] Cavalli E, Nicoletti GRP, Nicoletti F (2025). Historically based perspective on the immunotherapy of type 1 diabetes: where we have been, where we are, and where we may go. J Clin Med.

[53] Coutant F, Miossec P (2016). Altered dendritic cell functions in autoimmune diseases: distinct and overlapping profiles. Nat Rev Rheumatol.

[54] Jin F, Xie L, Zhang H, Fan X, Tian J, Liu W, Xiao Y (2025). Dendritic cells: origin, classification, development, biological functions, and therapeutic potential. MedComm (2020).

[55] Zhu J, Yamane H, Paul WE (2010). Differentiation of effector CD4 T cell populations (*). Annu Rev Immunol.

[56] Kolaczkowska E, Kubes P (2013). Neutrophil recruitment and function in health and inflammation. Nat Rev Immunol.

[57] Ip B, Cilfone NA, Belkina AC, DeFuria J, Jagannathan-Bogdan M, Zhu M, Kuchibhatla R (2016). Th17 cytokines differentiate obesity from obesity-associated type 2 diabetes and promote TNFalpha production. Obesity (Silver Spring).

[58] Zhu L, Song H, Zhang L, Meng H (2019). Characterization of IL-17-producing Treg cells in type 2 diabetes patients. Immunol Res.

[59] Chang YC, Hee SW, Chuang LM (2021). T helper 17 cells: A new actor on the stage of type 2 diabetes and aging?. J Diabetes Investig.

[60] Mahlangu T, Dludla PV, Nyambuya TM, Mxinwa V, Mazibuko-Mbeje SE, Cirilli I, Marcheggiani F (2020). A systematic review on the functional role of Th1/Th2 cytokines in type 2 diabetes and related metabolic complications. Cytokine.

[61] Antonia SJ (2019). Durvalumab after chemoradiotherapy in stage III non-small-cell lung cancer. Reply. N Engl J Med.

[62] Hellmann MD, Paz-Ares L, Bernabe Caro R, Zurawski B, Kim SW, Carcereny Costa E, Park K (2019). Nivolumab plus ipilimumab in advanced non-small-cell lung cancer. N Engl J Med.

[63] Sun YM, Wang Y, Sun XX, Chen J, Gong ZP, Meng HY (2020). Clinical efficacy of immune checkpoint inhibitors in older non-small-cell lung cancer patients: a meta-analysis. Front Oncol.

[64] Cressey R, Han MTT, Khaodee W, Xiyuan G, Qing Y (2024). Navigating PRKCSH's impact on cancer: from N-linked glycosylation to death pathway and anti-tumor immunity. Front Oncol.

[65] Daeron M (2022). The immune system as a system of relations. Front Immunol.

[66] Lv B, Wang Y, Ma D, Cheng W, Liu J, Yong T, Chen H (2022). Immunotherapy: Reshape the Tumor Immune Microenvironment. Front Immunol.

[67] Farhood B, Najafi M, Mortezaee K (2019). CD8(+) cytotoxic T lymphocytes in cancer immunotherapy: A review. J Cell Physiol.

